# Robust optimization model for uncertain multiobjective linear programs

**DOI:** 10.1186/s13660-018-1612-3

**Published:** 2018-01-18

**Authors:** Lei Wang, Min Fang

**Affiliations:** School of Economic Mathematics, Southwestern University of Finance and Economics, Chengdu, Sichuan 610074 P.R. China

**Keywords:** 90A14, 47H04, 47S40, multiobjective linear programs, robust efficient solutions, scalarization, uncertainty sets

## Abstract

In this paper, we consider the multiobjective linear programs where coefficients in the objective function belong to uncertainty sets. We introduce the concept of robust efficient solutions to uncertain multiobjective linear programming problems. By using two scalarization methods, the weighted sum method and the *ϵ*-constraint method, we obtain that the robust efficient solutions for uncertain multiobjective linear programs with ellipsoidal uncertainty sets and general norm uncertainty sets can be computed by some deterministic optimization problems.

## Introduction

The parameter values of optimization problems in real world are usually uncertain due to prediction errors, estimation errors, or lack of information at the time of decision. Therefore, it is important to solve such uncertain optimization problems for decision maker. In 1973, Soyster [1] first introduced the robust linear programs where coefficients are uncertain. The main idea is to assume that the coefficients can be any scenario in the uncertainty set and to find a solution that is feasible for all possible scenarios from the uncertainty set. The interest of robust optimization was revived in the 1990s (see, e.g., [2–10]). In 2009, Ben-Tal, El-Ghaoui, and Nemirovski [11] introduced a number of important results in robust linear optimization, robust conic optimization, and robust multistage optimization. Robust optimization has become a powerful approach to handle uncertain optimization problems.

On the other hand, the equilibrium problem provides a general mathematical model for a wide range of practical problems, such as optimization problems, Nash equilibria problems, fixed point problems, variational inequality problems, and complementarity problems, and has been investigated intensively. For more details, we refer to [12–15]. As a particular case of the vector equilibrium problem, multiobjective optimization problems arise in a large number of applications such as transportation, finance, communication, etc. Naturally, the issue of uncertain data affects single objective optimization problems in the same way as it affects these multiobjective ones. The essential problem in multiobjective optimization is to find the *Pareto efficient solutions*, meaning the feasible solutions such that no objective can be improved without sacrificing others; see, for example, Miettinen [16] and Ehrgott [17]. Therefore, in multiobjective optimization problems with data uncertainty, it is very important how to find *robust efficient solutions* that are less sensitive to small perturbations in variables.

In 2006, Deb and Gupta [18] presented two different robust multiobjective optimization procedures. The first one replaces all objective functions by their mean functions, and robust solution is defined as the efficient solution to the resulting deterministic optimization problem. The second one adds constraints to the predefined limit and optimizes the original objective functions. Recently, Kuroiwa and Lee [19] defined three kinds of robust efficient solutions, which are different from Deb and Gupta [18] for the uncertain multiobjective optimization problems. They also established necessary optimality theorems for robust efficient solutions and gave scalarization methods for robust efficient solutions of multiobjective optimization problems. Ehrgott, Ide, and Schobel [20] generalized the concept of minimax robustness introduced by Ben-Tal, El-Ghaoui, and Nemirovski [11] from single objective optimization problems to multiobjective optimization problems. They proposed robust Pareto efficiency and discussed how to find robust efficient solutions for uncertain multiobjective optimization problems. Goherna, Jeyakumar, Li, and Perez [21] introduced the definition of radius of robust feasibility and analyzed the robust weakly efficient solution of a multiobjective linear programming problems with data uncertainty both in the objective function and constraints. They also gave numerically tractable optimality conditions for highly robust weakly efficient solutions. Very recently, Bokrantz and Fredriksson [22] provided necessary and sufficient conditions for robust efficiency studied by Ehrgott, Ide, and Schobel [20] to multiobjective optimization problems with data uncertainty. They also applied these results to the field of therapy for cancer treatment.

Motivated by the works mentioned, in this paper, we consider the multiobjective linear programs where the coefficients $a_{i}$ and $b_{i}$ in the objective function belong to uncertain but bounded sets $U_{i}$. We introduce the concept of robust efficient solution to uncertain multiobjective linear programs (UMLPs). Also, we show that two scalarization methods, the weighted sum method and the *ϵ*-constraint method, can be used to find robust efficient solutions of UMLP with ellipsoidal uncertainty sets and general norm uncertainty sets. The structure of the paper is as follows. In Section 2, we introduce the uncertain multiobjective linear programming problems and the concept of robust efficient solution to UMLP. In Section 3, we give the ellipsoidal uncertainty sets and general norm uncertainty sets. Using the weighted sum method, we show that the robust efficient solution of UMLP can be found by some deterministic optimization problems. In Section 4, we use the *ϵ*-constraint method to compute the robust efficient solution of UMLP under ellipsoidal uncertainty sets and general norm uncertainty sets. Finally, we conclude in Section 5.

## Introduction to uncertain multiobjective linear programs

### Deterministic multiobjective linear programs

Consider the following multiobjective linear programming problem:

MLP $$\begin{aligned}& \begin{aligned}&\min f(x)= \bigl(a^{T}_{1}x+b_{1}, a^{T}_{2}x+b_{2}, \ldots, a^{T}_{m}x+b _{m} \bigr) \\ & \mbox{s.t.}\quad x\in X, \end{aligned} \end{aligned}$$

where $f:R^{n}\rightarrow R^{m}$, $X\subseteq R^{n}$, and $(a_{i}, b _{i})\in R^{n}\times R$, $i=1, 2,\ldots, m$, are coefficients.

In this paper, we use the order relation ⪰ (see Ehrgott [17]): For $y^{1}, y^{2}\in R^{m}$, we write $y^{1}\succeq y^{2}$ if $y^{1}$ is greater than or equal to $y^{2}$ in every component and greater in at least one component. Furthermore, we define the cone $R^{m}_{\succeq }=\{x\in R^{m}: x\succeq 0\}$.

The Pareto efficient solution to (MLP) is defined as follows:

#### Definition 1

A feasible solution $x^{*}\in X$ to (MLP) is Pareto efficient if there is no feasible solution $x\in X$ such that $f(x)\in f(x^{*})-R^{m}_{\succeq }$.

### Robust multiobjective linear programs

Consider the following uncertain multi-objective linear programming problem

UMLP $$\begin{aligned}& \begin{aligned}&\min f(x; \tilde{a}_{i}, \tilde{b}_{i})= \bigl( \tilde{a}^{T}_{1}x+ \tilde{b}_{1}, \tilde{a}^{T}_{2}x+ \tilde{b}_{2}, \ldots, \tilde{a} ^{T}_{m}x+ \tilde{b}_{m} \bigr) \\ &\mbox{s.t.}\quad x\in X, \end{aligned} \end{aligned}$$

where the coefficients $(\tilde{a}_{i},\tilde{b}_{i})$, $i=1, \ldots, m$, are uncertain and belong to the bounded uncertainty set $U_{i}$, $i=1,2,\ldots,m$, and set $U=\Pi^{m}_{i=1} U_{i}$.

Given an uncertain multiobjective linear programming problem (UMLP), there arises the same question of how to find feasible solutions $x \in X$ as in a single objective optimization problem. We cannot take the worst case under all scenarios for evaluating solutions because in multiobjective optimization problems, we obtain that the objective value for each scenario is a vector. Therefore, let $f_{U}(x)=\{f(x; \tilde{a}_{i},\tilde{b}_{i}):(\tilde{a}_{i},\tilde{b}_{i})\in U_{i}, i=1,2,\ldots, m\}\subset R^{m}$. We can generalize the concept of efficiency as given in Definition 1 with this notion.

#### Definition 2

A feasible solution $x^{*}\in X$ to problem (UMLP) is robust efficient if there is no feasible solution $x\in X\setminus \{x^{*}\}$ such that $f_{U}(x)\subseteq f_{U}(x^{*})-R^{m}_{\succeq }$.

Thus, all possible objective values of a solution $x^{*}$ are considered over all scenarios, namely the set $f_{U}(x^{*})$.

#### Example 1

**Figure 1:**
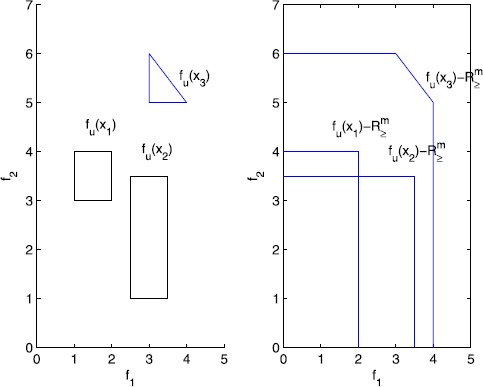
**Illustration of robust efficient solution.**

Consider an uncertain multiobjective linear programming problem with two objectives $f_{1}=\tilde{a}^{T}_{1}x+\tilde{b}_{1}$ and $f_{2}=\tilde{a}^{T}_{2}x+\tilde{b}_{2}$. The left picture in Figure 1 refers to (UMLP) with feasible set $X=\{x_{1}, x_{2}, x_{3}\}$, and the three sets $f_{U}(x_{1})$, $f_{U}(x_{2})$, $f_{U}(x_{3})$ are polyhedrons. In the right picture, we can see that none of $f_{U}(x _{1})-R_{\geq }^{m}$ and $f_{U}(x_{2})-R_{\geq }^{m}$ contains any other set $f_{U}(x_{i})$, and thus $x_{1}$ and $x_{2}$ are both robust efficient. On the other hand, $f_{U}(x_{3})-R_{\geq }^{m}$ contains $f_{U}(x_{1})$ and $f_{U}(x_{2})$, and thus $x_{3}$ is not robust efficient.

Having introduced the definition of robust efficient solutions for uncertain multiobjective linear programs (UMLPs), in Sections 3 and 4, we use two scalarization methods, the weighted sum method and the *ϵ*-constraint method, to find the robust efficient solution for (UMLP).

## Weighted sum scalarization

Weighted sum scalarization is the most common approach to evaluate efficient solutions for a deterministic multiobjective optimization problem. The weighted sum problem (WSP) for a given deterministic multiobjective linear programming problem is given as follows:

WSP $$\begin{aligned}& \begin{aligned}&\min \sum_{i=1}^{m} \lambda_{i} \bigl(a^{T}_{i}x+b_{i} \bigr) \\ &\mbox{s.t.}\quad \sum_{i=1}^{m} \lambda_{i}=1, \\ &\hphantom{\mbox{s.t.}\quad }\lambda_{i}\geq 0, i=1,2,\ldots,m, \\ &\hphantom{\mbox{s.t.}\quad } x\in X. \end{aligned} \end{aligned}$$

We now use the weighted sum scalarization method to reduce an uncertain multiobjective linear programming problem to a single objective uncertain linear programming problem in order to compute robust efficient solutions by computing robust optimal solutions for the uncertain single objective linear programming problem. Next, we introduce the robust counterpart of the weighted sum scalarization problem of an uncertain multiobjective linear programs (RWSPs) as:

RWSP $$\begin{aligned}& \begin{aligned}&\min \max_{(\tilde{a}_{i},\tilde{b}_{i})\in U_{i}}\sum _{i=1}^{m} \lambda_{i} \bigl( \tilde{a}^{T}_{i}x+ \tilde{b}_{i} \bigr) \\ &\mbox{s.t.}\quad \sum_{i=1}^{m} \lambda_{i}=1, \\ &\hphantom{\mbox{s.t.}\quad } \lambda_{i}\geq 0, i=1,2,\ldots,m, \\ &\hphantom{\mbox{s.t.}\quad } x\in X. \end{aligned} \end{aligned}$$

### Lemma 1

*Given an uncertain multiobjective linear programming problem* (UMLP), *if* $x^{*}\in X$ *is the unique optimal solution to* (RWSP), *then* $x^{*}$ *is robust efficient solution for* (UMLP).

### Proof

Assum that $x^{*}$ is not robust efficient solution for (UMLP). Then there exists *x̂* such that $f_{U}(\hat{x})\in f _{U}(x^{*})-R^{m}_{\succeq }$. This implies that, for all $(\tilde{a} _{i},\tilde{b}_{i})\in U_{i}$, there exists $(\tilde{\alpha }_{i}, \tilde{\beta }_{i})\in U_{i}$ such that

$$\tilde{a}_{i}^{T}\hat{x}+\tilde{b}_{i}\leq \tilde{\alpha }_{i}^{T}x ^{*}+\tilde{\beta }_{i},\quad \forall i=1,2,\ldots, m. $$

Now choose arbitrary but fixed $\lambda_{i}\geq 0$, $i=1,2,\ldots,m$, such that $\sum_{i=1}^{m}\lambda_{i}=1$. We have

$$\sum_{i=1}^{m}\lambda_{i} \bigl( \tilde{a}_{i}^{T}\hat{x}+\tilde{b}_{i} \bigr) \leq \sum_{i=1}^{m}\lambda_{i} \bigl( \tilde{\alpha }_{i}^{T}x^{*}+ \tilde{\beta }_{i} \bigr)\leq \max_{(\tilde{\alpha }_{i}',\tilde{\beta }_{i}')\in U_{i}}\sum _{i=1} ^{m}\lambda_{i} \bigl(\tilde{\alpha }_{i}^{\prime\, T}x^{*}+\tilde{\beta }_{i}' \bigr) $$

for all $(\tilde{a}_{i},\tilde{b}_{i})\in U_{i}$. This implies that

$$\max_{(\tilde{a}_{i}',\tilde{b}_{i}')\in U_{i}}\sum_{i=1}^{m} \lambda _{i} \bigl(\tilde{a}_{i}^{\prime\, T}\hat{x}+ \tilde{b}_{i}' \bigr)\leq \max_{(\tilde{\alpha }_{i}',\tilde{\beta }_{i}')\in U_{i}}\sum _{i=1} ^{m}\lambda_{i} \bigl( \tilde{\alpha }_{i}^{\prime\, T}x^{*}+\tilde{\beta }_{i}' \bigr), $$

which contradicts the fact that $x^{*}$ is the unique optimal solution to (RWSP). □

We further show that robust efficient solutions for uncertain multiobjective linear programming problems with ellipsoidal uncertainty sets and general uncertainty sets can be found by solving deterministic optimization problems using weighted sum scalarization and thus can be computed by existing technology of deterministic optimization problems.

### Ellipsoidal uncertainty sets

Consider the ellipsoidal uncertainty sets

U1 $$\begin{aligned}& U_{i}= \Biggl\{ (\tilde{a}_{i}, \tilde{b}_{i})= \bigl(a_{i}^{0}, b_{i}^{0} \bigr)+\sum_{j=1} ^{l}u^{j} \bigl(a_{i}^{j}, b_{i}^{j} \bigr)\vert \Vert u\Vert _{2}\leq 1 \Biggr\} , \end{aligned}$$

where $i=1, 2,\ldots, m$, $(a_{i}^{0}, b_{i}^{0})$ are nominal values of (RWSP), $(a_{i}^{j}, b_{i}^{j})$ are the given directions of perturbation, and $u^{j}$ are the uncertain variables with $\Vert u\Vert _{2} \leq 1$.

#### Theorem 1

*Consider the uncertain multiobjective linear programming problem* (UMLP) *with ellipsoidal uncertainty sets* $U_{i}$ *given as in* (U1). *If* $x^{*}$ *is the unique optimal solution to the deterministic second*-*order cone programming*

DP1 $$\begin{aligned}& \begin{aligned}&\min \sum_{i=1}^{m} \lambda_{i} \bigl\{ \bigl(a^{0T}_{i}x+b_{i}^{0} \bigr)+\Vert A_{i}x+B_{i}\Vert _{2} \bigr\} \\ &\textit{s.t.}\quad \sum_{i=1}^{m} \lambda_{i}=1, (\mbox{)} \\ &\hphantom{\textit{s.t.}\quad } \lambda_{i}\geq 0, i=1,2,\ldots,m, \\ &\hphantom{\textit{s.t.}\quad } x\in X, \end{aligned} \end{aligned}$$

*where* $A_{i}x+B_{i}=(a_{i}^{1T}x+b_{i}^{1}, a_{i}^{2T}x+b_{i}^{2}, \ldots, a_{i}^{lT}x+b_{i}^{l})^{T}$, *then* $x^{*}$ *is a robust efficient solution for* (UMLP).

#### Proof

First, we show that (RWSP) with ellipsoidal uncertainty sets (U1) is equivalent to (DP1). For *x* fixed, the worst-case residual of objective function in (RWSP) with ellipsoidal uncertainty sets (U1) can be rewritten as

$$\begin{aligned}& \max_{(\tilde{a}_{i},\tilde{b}_{i})\in U_{i}}\sum_{i=1}^{m} \lambda _{i} \bigl(\tilde{a}^{T}_{i}x+ \tilde{b}_{i} \bigr) \\& \quad =\max_{\Vert u\Vert _{2}\leq 1}\sum_{i=1}^{m} \lambda_{i} \Biggl\{ \bigl(a_{i}^{0T}x+b _{i}^{0} \bigr)+\sum_{j=1}^{l}u_{j} \bigl(a_{i}^{jT}x+b_{i}^{j} \bigr) \Biggr\} \\& \quad =\sum_{i=1}^{m}\lambda_{i} \Biggl\{ \bigl(a_{i}^{0T}x+b_{i}^{0} \bigr)+ \max_{\Vert u\Vert _{2}\leq 1}\sum_{j=1}^{l}u_{j} \bigl(a_{i}^{jT}x+b_{i}^{j} \bigr) \Biggr\} . \end{aligned}$$

Let $A_{i}x+B_{i}=(a_{i}^{1T}x+b_{i}^{1}, a_{i}^{2T}x+b_{i}^{2}, \ldots, a_{i}^{lT}x+b_{i}^{l})^{T}$. Then we have

$$\begin{aligned}& \sum_{i=1}^{m}\lambda_{i} \Biggl\{ \bigl(a_{i}^{0T}x+b_{i}^{0} \bigr)+ \max_{\Vert u\Vert _{2}\leq 1}\sum_{j=1}^{l}u_{j} \bigl(a_{i}^{jT}x+b_{i}^{j} \bigr) \Biggr\} \\& \quad =\sum_{i=1}^{m}\lambda_{i} \Bigl\{ \bigl(a_{i}^{0T}x+b_{i}^{0} \bigr)+ \max_{\Vert u\Vert _{2}\leq 1} \bigl\Vert u^{T}(A_{i}x+B_{i}) \bigr\Vert _{2} \Bigr\} \\& \quad = \sum_{i=1}^{m}\lambda_{i} \bigl\{ \bigl(a_{i}^{0T}x+b_{i}^{0} \bigr)+\Vert A_{i}x+B_{i}\Vert _{2} \bigr\} . \end{aligned}$$

This implies that (RWSP) with ellipsoidal uncertainty sets (U1) is equivalent to (DP1). By Lemma 1 we derive that if $x^{*}$ is the unique optimal solution to (DP1), then $x^{*}$ is a robust efficient solution for (UMLP). □

### General norm uncertainty sets

Consider the general norm uncertainty sets

U2 $$\begin{aligned}& U_{i}= \bigl\{ (\tilde{a}_{i}, \tilde{b}_{i})= \bigl(a_{i}^{0}, b_{i}^{0} \bigr)+(\Delta a_{i}, \Delta b_{i}) \vert \bigl\Vert M(\Delta a_{i}, \Delta b_{i})^{T} \bigr\Vert \leq \delta \bigr\} , \end{aligned}$$

where *M* is an $n\times 1$ invertible matrix, *δ* is a given positive constant, and $\Vert \cdot \Vert $ is a general norm. Given a general norm $\Vert x\Vert $ for a real vector *x*, its dual norm is given by

$$\Vert z\Vert ^{*}=\max_{\Vert x\Vert \leq 1}z^{T}x. $$

#### Theorem 2

*Consider the uncertain multiobjective linear programming problem* (UMLP) *with general norm uncertainty sets* $U_{i}$ *given as in* (U2). *If* $x^{*}$ *is the unique optimal solution to the deterministic optimization problem*

DP2 $$\begin{aligned}& \begin{aligned}&\min \sum_{i=1}^{m} \lambda_{i} \bigl\{ \bigl(a^{0T}_{i}x+b_{i}^{0} \bigr)+\delta \bigl\Vert M^{-1}(x, 1)^{T} \bigr\Vert ^{*} \bigr\} \\ &\textit{s.t.}\quad \sum_{i=1}^{m} \lambda_{i}=1, \\ &\hphantom{\textit{s.t.}\quad } \lambda_{i}\geq 0, i=1,2,\ldots,m, \\ &\hphantom{\textit{s.t.}\quad } x\in X, \end{aligned} \end{aligned}$$

*then* $x^{*}$ *is a robust efficient solution for* (UMLP).

#### Proof

First, we show that (RWSP) with general norm uncertainty sets (U2) is equivalent to (DP2). For *x* fixed, the worst-case residual of objective function in (RWSP) with general norm uncertainty sets (U2) can be rewritten as

$$\begin{aligned}& \max_{(\tilde{a}_{i},\tilde{b}_{i})\in U_{i}}\sum_{i=1}^{m} \lambda _{i} \bigl(\tilde{a}^{T}_{i}x+ \tilde{b}_{i} \bigr) \\& \quad =\max_{\Vert M(\Delta a_{i}, \Delta b_{i})^{T}\Vert \leq \delta }\sum_{i=1} ^{m}\lambda_{i} \bigl\{ \bigl(a_{i}^{0T}x+b_{i}^{0} \bigr)+ \bigl(\Delta a_{i}^{T}x+\Delta b _{i} \bigr) \bigr\} \\& \quad =\sum_{i=1}^{m}\lambda_{i} \Bigl\{ \bigl(a_{i}^{0T}x+b_{i}^{0} \bigr)+ \max_{\Vert M(\Delta a_{i}, \Delta b_{i})^{T}\Vert \leq \delta }(\Delta a_{i}, \Delta b_{i}) (x, 1)^{T} \Bigr\} \\& \quad =\sum_{i=1}^{m}\lambda_{i} \Bigl\{ \bigl(a_{i}^{0T}x+b_{i}^{0} \bigr)+\max_{y\leq 1}y ^{T}\delta M^{-1}(x, 1)^{T} \Bigr\} \\& \quad = \sum_{i=1}^{m}\lambda_{i} \bigl\{ \bigl(a^{0T}_{i}x+b_{i}^{0} \bigr)+\delta \bigl\Vert M^{-1}(x, 1)^{T} \bigr\Vert ^{*} \bigr\} . \end{aligned}$$

This implies that (RWSP) with general norm uncertainty sets (U2) is equivalent to (DP2). By Lemma 1 we derive that if $x^{*}$ is the unique optimal solution to (DP2), then $x^{*}$ is a robust efficient solution for (UMLP). □

#### Remark

1. If the general norm uncertainty set is given by the Euclidean norm $\Vert \cdot \Vert _{2}$, then (DP2) can be formulated as a second-order cone optimization problem because the Euclidean norm is self-dual.
2. If the general norm uncertainty set is described by either $\Vert \cdot \Vert _{1}$ or $\Vert \cdot \Vert _{\infty }$, then (DP2) can be formulated as a linear programming problem.
3. We consider the uncertainty set described by the D-norm was studied by Bertsimas and Sim [4–6]. The D-norm of $y\in R^{n}$ is given as follows:
  $$\Vert y \Vert _{p}=\max_{ \{S\cup {t}\vert S\subseteq N, \vert S\vert \le \lfloor p\rfloor,t\in N\setminus S \}} \biggl\{ \sum _{j\in S}\vert y_{j}\vert + \bigl(p- \lfloor p \rfloor \bigr)\vert y_{t}\vert \biggr\} , $$

where $p\in [1,n]$ is the maximum number of variables, and *p* is not necessarily integer. In other words, *p* is a parameter used for controlling the degree of conservatism of the solution for (RWSP). Speaking directly, it is unlike that all of the $y_{j}$, $j=1,2,\ldots,n$, will change. The dual norm of the norm $\Vert \cdot \Vert _{p}$ is given by

$$\Vert y\Vert ^{*}_{p}=\max \bigl(\Vert y\Vert _{\infty }, \Vert y\Vert _{1}/p \bigr). $$

By Theorem 2 we can obtain that if the general norm uncertainty set is given by the D-norm, then (DP2) can also be formulated as a linear programming problem.

## *ϵ*-constraint scalarization

*ϵ*-constraint scalarization is another approach for evaluating efficient solutions for deterministic multiobjective optimization problems. For arbitrary $t\in \{1,2,\ldots,m\}$ and a parameter vector $\epsilon \in R^{m}$, the *ϵ*-constraint linear programming problem is defined as

ECP $$\begin{aligned}& \begin{aligned}&\min a_{t}^{T}x+b_{t} \\ &\mbox{s.t.}\quad a_{k}^{T}x+b_{k}\leq \epsilon_{k},\quad \forall k\in \{1,2,\ldots,m\}\backslash \{t\}, \\ &\hphantom{\mbox{s.t.}\quad } x\in X. \end{aligned} \end{aligned}$$

Note that, the problem (ECP) does not depend on the parameter $\epsilon_{k}$. We now extend the *ϵ*-constraint method by reducing an uncertain multiobjective linear programming problem to a single objective uncertain linear programming problem. Therefore, we define the robust counterpart of the *ϵ*-constraint scalarization problem of an uncertain multiobjective linear programming problem as follows:

RECP $$\begin{aligned}& \begin{aligned}&\min \max_{(\tilde{a}_{t},\tilde{b}_{t})\in U_{t}} \tilde{a}^{T} _{t}x+ \tilde{b}_{t} \\ &\mbox{s.t.}\quad \max_{(\tilde{a}_{k},\tilde{b}_{k})\in U_{k}} \tilde{a}^{T} _{k}x+ \tilde{b}_{k}\leq \epsilon_{k}, \quad \forall k\in \{1,2,\ldots,m \}\backslash \{t\}, \\ &\hphantom{\mbox{s.t.}\quad } x\in X. \end{aligned} \end{aligned}$$

### Lemma 2

*Given an uncertain multiobjective linear programming problem* (UMLP), *if* $x^{*}\in X$ *is the unique optimal solution to* (RECP) *for some* $\epsilon \in R^{m}$ *and some* $t\in \{1,2,\ldots, m\}$, *then* $x^{*}$ *is a robust efficient solution for* (UMLP).

### Proof

Assume that $x^{*}$ is not a robust efficient solution for (UMLP). Then there exists *x̂* such that $f_{U}(\hat{x})\in f _{U}(x^{*})-R^{m}_{\succeq }$. This implies that, for all $(\tilde{a} _{i},\tilde{b}_{i})\in U_{i}$, there exists $(\tilde{\alpha }_{i}, \tilde{\beta }_{i})\in U_{i}$ such that

$$\tilde{a}_{i}^{T}\hat{x}+\tilde{b}_{i}\leq \tilde{\alpha }_{i}^{T}x ^{*}+\tilde{\beta }_{i}, \quad \forall i=1,2,\ldots, m. $$

Then, in the constraints of (RECP), we obtain

$$\max_{(\tilde{a}_{k}',\tilde{b}_{k}')\in U_{k}}\tilde{a}_{k}^{\prime\, T} \hat{x}+ \tilde{b}_{k}'\leq \tilde{\alpha }_{k}^{T}x^{*}+ \tilde{\beta }_{k}\leq \max_{(\tilde{\alpha }_{k}',\tilde{\beta }_{k}')\in U_{k}} \tilde{\alpha }_{k}^{\prime\, T}x^{*}+\tilde{\beta }_{k}' \leq \epsilon_{k},\quad \forall k\in \{1,2,\ldots,m\}\backslash \{t\} . $$

On the other hand, in the objective function of (RECP), we have

$$\max_{(\tilde{a}_{t}',\tilde{b}_{t}')\in U_{t}}\tilde{a}_{t}^{\prime\, T} \hat{x}+ \tilde{b}_{t}'\leq \tilde{\alpha }_{t}^{T}x^{*}+ \tilde{\beta }_{t}\leq \max_{(\tilde{\alpha }_{t}',\tilde{\beta }_{t}')\in U_{t}} \tilde{\alpha }_{t}^{\prime\, T}x^{*}+\tilde{\beta }_{t}'. $$

But then *x̂* is feasible for (RECP) and has an equal or better objective value than $x^{*}$. This is a contradiction to the assumption that $x^{*}$ is the unique optimal solution to (RECP). □

Next, we show that robust efficient solutions for uncertain multiobjective linear programming problems with ellipsoidal uncertainty sets and general uncertainty sets can be found by solving deterministic optimization problems using *ϵ*-constraint scalarization and so can be computed by existing technology of deterministic optimization problems.

### Theorem 3

*Consider the uncertain multiobjective linear programming problem* (UMLP) *with ellipsoidal uncertainty sets* $U_{i}$ *given as in* (U1). *If* $x^{*}$ *is the unique optimal solution to the deterministic second*-*order cone programming*

DP3 $$\begin{aligned}& \begin{aligned}&\min a_{t}^{0T}x+b_{t}^{0}+\Vert A_{t}x+B_{t}\Vert _{2} \\ &\textit{s.t.}\quad a_{k}^{0T}x+b_{k}^{0}+\Vert A_{k}x+B_{k}\Vert _{2}\leq \epsilon_{k}, \quad \forall k\in \{1,2,\ldots,m\}\backslash \{t\}, \\ &\hphantom{\textit{s.t.}\quad } x\in X, \end{aligned} \end{aligned}$$

*where* $A_{i}x+B_{i}=(a_{i}^{1T}x+b_{i}^{1}, a_{i}^{2T}x+b_{i}^{2}, \ldots, a_{i}^{lT}x+b_{i}^{l})^{T}$, $i=1,2,\ldots,m$, *then* $x^{*}$ *is a robust efficient solution for* (UMLP).

### Proof

First, we show that (RECP) with ellipsoidal uncertainty sets (U1) is equivalent to (DP3). For fixed *x*, the worst-case residual of objective function in (RECP) with ellipsoidal uncertainty sets (U1) can be rewritten as

$$\begin{aligned}& \max_{(\tilde{a}_{t},\tilde{b}_{t})\in U_{t}}\tilde{a}^{T}_{t}x+ \tilde{b}_{t} \\& \quad =\max_{\Vert u\Vert _{2}\leq 1} \Biggl\{ \bigl(a_{t}^{0T}x+b_{t}^{0} \bigr)+\sum_{j=1}^{l}u _{j} \bigl(a_{t}^{jT}x+b_{t}^{j} \bigr) \Biggr\} \\& \quad = a_{t}^{0T}x+b_{t}^{0}+\max _{\Vert u\Vert _{2}\leq 1}\sum_{j=1}^{l}u_{j} \bigl(a _{t}^{jT}x+b_{t}^{j} \bigr). \end{aligned}$$

Let $A_{t}x+B_{t}=(a_{t}^{1T}x+b_{t}^{1}, a_{t}^{2T}x+b_{t}^{2}, \ldots, a_{t}^{lT}x+b_{t}^{l})^{T}$. Then we have

$$\begin{aligned}& a_{t}^{0T}x+b_{t}^{0}+\max _{\Vert u\Vert _{2}\leq 1}\sum_{j=1}^{l}u_{j} \bigl(a _{t}^{jT}x+b_{t}^{j} \bigr) \\& \quad =a_{t}^{0T}x+b_{t}^{0}+\max _{\Vert u\Vert _{2}\leq 1} \bigl\Vert u^{T}(A_{t}x+B_{t}) \bigr\Vert _{2} \\& \quad = a_{t}^{0T}x+b_{t}^{0}+\Vert A_{t}x+B_{t}\Vert _{2}. \end{aligned}$$

Using a similar approach in the objective function, we can derive that the worst-case of constraints in (RECP) can be rewritten as

$$\max_{(\tilde{a}_{k},\tilde{b}_{k})\in U_{k}}\tilde{a}^{T}_{k}x+ \tilde{b}_{k}= a_{k}^{0T}x+b_{k}^{0}+ \Vert A_{k}x+B_{k}\Vert _{2}\leq \epsilon _{k},\quad \forall k\in \{1,2,\ldots,m\}\backslash \{t\}. $$

From the previous conclusions we have that (RECP) with ellipsoidal uncertainty sets is equivalent to (DP3). By Lemma 2 we derive that if $x^{*}$ is the unique optimal solution to (DP3), then $x^{*}$ is a robust efficient solution for (UMLP). □

### Theorem 4

*Consider the uncertain multiobjective linear programming problem* (UMLP) *with general norm uncertainty sets* $U_{i}$ *given as in* (U2). *If* $x^{*}$ *is the unique optimal solution to the deterministic optimization problem*

DP4 $$\begin{aligned}& \begin{aligned}&\min a^{0T}_{t}x+b_{t}^{0}+\delta \bigl\Vert M^{-1}(x, 1)^{T} \bigr\Vert ^{*} \\ &\textit{s.t.}\quad a^{0T}_{k}x+b_{k}^{0}+\delta \bigl\Vert M^{-1}(x, 1)^{T} \bigr\Vert ^{*}\leq \epsilon_{k}, \quad \forall k\in \{1,2,\ldots,m\}\backslash \{t\}, \\ &\hphantom{\textit{s.t.}\quad } x\in X, \end{aligned} \end{aligned}$$

*then* $x^{*}$ *is a robust efficient solution for* (UMLP).

### Proof

First, we show that (RECP) with general norm uncertainty sets (U2) is equivalent to (DP4). For fixed *x*, the worst-case residual of objective function in (RECP) with general norm uncertainty sets (U2) can be rewritten as

$$\begin{aligned}& \max_{(\tilde{a}_{t},\tilde{b}_{t})\in U_{i}}\tilde{a}^{T}_{t}x+ \tilde{b}_{t} \\& \quad =a_{t}^{0T}x+b_{t}^{0}+ \max _{\Vert M(\Delta a_{t}, \Delta b_{t})^{T}\Vert \leq \delta }(\Delta a_{t}, \Delta b_{t}) (x, 1)^{T} \\& \quad =a_{t}^{0T}x+b_{t}^{0}+\max _{y\leq 1}y^{T}\delta M^{-1}(x, 1)^{T} \\& \quad = a^{0T}_{t}x+b_{t}^{0}+\delta \bigl\Vert M^{-1}(x, 1)^{T} \bigr\Vert ^{*}. \end{aligned}$$

Using a similar approach in the objective function, we can derive that the worst-case of constraints in (RECP) can be rewritten as

$$\max_{(\tilde{a}_{k},\tilde{b}_{k})\in U_{k}}\tilde{a}^{T}_{k}x+ \tilde{b}_{k}= a^{0T}_{k}x+b_{k}^{0}+ \delta \bigl\Vert M^{-1}(x, 1)^{T} \bigr\Vert ^{*} \leq \epsilon_{k}, \quad \forall k\in \{1,2,\ldots,m\} \backslash \{t \}. $$

From the previous conclusions we have that (RECP) with general norm uncertainty sets is equivalent to (DP4). By Lemma 2, we derive that if $x^{*}$ is the unique optimal solution to (DP4), then $x^{*}$ is a robust efficient solution for (UMLP). □

### Remark

1. If the general norm uncertainty set is given by the Euclidean norm $\Vert \cdot \Vert _{2}$, then (DP4) can be formulated as a second-order cone programming with second-order cone constraints.
2. If the general norm uncertainty set is given by either $\Vert \cdot \Vert _{1}$ or $\Vert \cdot \Vert _{\infty }$, then (DP4) can be formulated as a linear programming problem.
3. If the general norm uncertainty set is described by the D-norm $\Vert \cdot \Vert _{p}$, then (DP4) can also be formulated as a linear programming problem.

## Conclusions

Multiobjective optimization and robust optimization have been well studied, but they are rarely considered in combination. In this paper, we consider the multiobjective linear programs where coefficients in the objective function belong to uncertain-but-bounded sets. First, we introduce the concept of a robust efficient solution to uncertain multiobjective linear programs. We also introduce two common scalarization methods, the weighted sum scalarization and *ϵ*-constraint scalarization, to compute robust efficient solutions of uncertain multiobjective linear programs. Finally, we obtain that the robust efficient solutions of uncertain multiobjective linear programs can be computed by some deterministic optimization problems using both weighted sum method and *ϵ*-constraint method.
